# Supplementary material for: Identification of photoperiod-induced specific miRNAs in the adrenal glands of Sunite sheep (Ovis aries)
Source: Front Vet Sci. 2022 Jul 22;9:888207. doi: 10.3389/fvets.2022.888207 (PMC9354845; doi:10.3389/fvets.2022.888207)
Supplement: Supplementary file 1 [file Data_Sheet_1.ZIP › Supplementary file/miRDeep2 file.zip!/miRDeep2 file/Know_Structure/LP42A3/oar-let-7g.pdf]

miRBase precursor : oar-let-7g
Total read count : 666643
oar-let-7g read count : 666623
remaining reads : 20

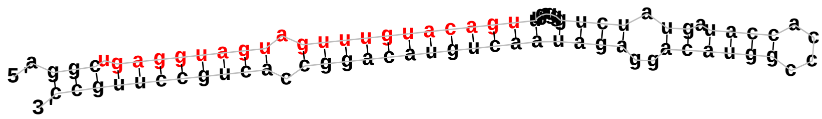

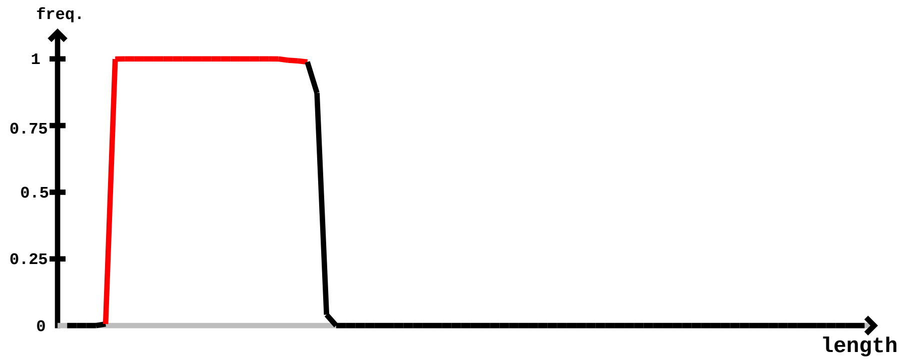

oar-let-7g

```
5'- aggcugagguaguaguuuguacaguuugagggucuaugauaccacccgguacaggagauaacuguacaggccacugccuugcc   -3'    exp
    .(((.((((((((.((((((((((((.....((((.((.((((....))))))..)))))))))))))))).)))))))))))    reads     mm     sample
    ...cugagguaguaguuugua..............................................................    2         0      seq
    ...cugagguaguaguuuguac.............................................................    23        0      seq
    ...cugagguaguaguuuguaca............................................................    19        0      seq
    ...cugagguaguaguuuguacag...........................................................    21        0      seq
    ...cugagguaguaguuuguacagu..........................................................    693       0      seq
    ...cugagguaguaguuuguacaguu.........................................................    2983      0      seq
    ...cugagguaguaguuuguacaguuu........................................................    120       0      seq
    ....ugagguagCaguuugua..............................................................    1         1      seq
    ....ugagguaguaguuugua..............................................................    102       0      seq
    ....ugaggAaguaguuugua..............................................................    1         1      seq
    ....ugagguaguaguuuguac.............................................................    3226      0      seq
    ....ugagguagCaguuuguac.............................................................    5         1      seq
    ....ugagguaguaguuuguaca............................................................    1572      0      seq
    ....ugagguaguaguuuguacag...........................................................    2654      0      seq
    ....ugagguaguaguuuguacagu..........................................................    76767     0      seq
    ....ugagguaguaguuuguacaguu.........................................................    551034    0      seq
    ....ugagguaguaguuuguacaguuu........................................................    26977     0      seq
    ....ugagguaguaguuuguacaguuug.......................................................    17        0      seq
    ....ugagguaguaguuuguacaguuuga......................................................    1         0      seq
    .....gagguaguaguuuguac.............................................................    1         0      seq
    .....gagguaguaguuuguaca............................................................    2         0      seq
    .....gagguaguaguuuguacag...........................................................    3         0      seq
    .....gagguaguaguuuguacagu..........................................................    50        0      seq
    .....gagguaguaguuuguacaguu.........................................................    321       0      seq
    .....gagguaguaguuuguacaguuu........................................................    23        0      seq
    ......agguaguaguuuguacaguu.........................................................    4         0      seq
    .......gguaguaguuuguacaguu.........................................................    1         0      seq
    ...........................................................aacuguacaggccacugccuug..    1         0      seq
    ............................................................acuguacaggccacugccuugc.    2         0      seq
    .............................................................cuguacaggccacugccuugc.    7         0      seq
    .............................................................cuguacaggccacugccuugcc    10        0      seq
```
